# Cardiologist-Directed Sedation Management in Patients Undergoing Transvenous Lead Extraction: A Single-Centre Retrospective Analysis

**DOI:** 10.3390/jcm12154900

**Published:** 2023-07-26

**Authors:** Matthias Bock, Matthew O’Connor, Amir Chouchane, Philip Schmidt, Claudia Schaarschmidt, Katharina Knoll, Fabian Bahlke, Florian Englert, Theresa Storz, Marc Kottmaier, Teresa Trenkwalder, Tilko Reents, Felix Bourier, Marta Telishevska, Sarah Lengauer, Gabriele Hessling, Isabel Deisenhofer, Christof Kolb, Carsten Lennerz

**Affiliations:** 1 German Heart Centre Munich, Department of Electrophysiology, Technical University of Munich, 80636 Munich, Germanychristof.kolb@web.de (C.K.); 2 DZHK (German Centre for Cardiovascular Research, Partner Site Munich, Heart Alliance), 80336 Munich, Germany; 3 Auckland City Hospital, Department of Cardiology, Auckland 1023, New Zealand

**Keywords:** deep sedation, cardiologist-directed deep sedation, transvenous lead extraction, lead revision, cardiac implantable electronic devices

## Abstract

Background: The demand for transvenous lead extraction (TLE) has increased. In line with this, the safety of such procedures has also increased. Traditionally, TLE is performed under resource-intensive general anaesthesia. This study aims to evaluate the safety and outcomes of Cardiologist-lead deep sedation for TLE. Methods: We retrospectively analysed 328 TLE procedures performed under deep sedation from 2016 to 2019. TLE procedures were performed by experienced electrophysiologists. Sedation was administered by a specifically trained cardiologist (bolus midazolam/fentanyl and propofol infusion). Procedural sedation data including blood pressure, medication administration and sedation time were collected. Complications related to sedation and the operative component of the procedure were analysed retrospectively. Results: The sedation-associated complication rate during TLE was 22.0%. The most common complication (75% of complications) was hypotension requiring noradrenaline, followed by bradycardia requiring atropine (13% of complications). Additionally, the unplanned presence of an anaesthesiologist was needed in one case (0.3%). Deep sedation was achieved with midazolam (mean dose 42.9 ± 26.5 µg/kg), fentanyl (mean dose 0.4 ± 0.6 µg/kg) and propofol (mean dose 3.5 ± 1.2 mg/kg/h). There was no difference in medication dosage between those with a sedation-associated complication and those without. Sedation-associated complications appeared significantly more in patients with reduced LVEF (*p* = 0.01), renal impairment (*p* = 0.01) and a higher American Society of Anaesthesiologists (ASA) class (*p* = 0.01). Conclusion: Deep sedation for TLE can be safely performed by a specifically trained cardiologist, with a transition to general anaesthesia required in only 0.3% of cases. We continue to recommend the on-call availability of an anaesthesiologist and cardiac surgeon in case of major complications.

## 1. Introduction

As the number of cardiac implantable electronic device (CIED) implantations is rising worldwide, the incidence of device-related complications is increasing too [1,2]. Leads are the Achilles’ heel of CIEDs, and lead dysfunction is a common reason for requiring interventional revision, often with transvenous lead extractions (TLE) [3,4,5]. Infections such as pocket infections and CIED-related endocarditis are a class 1 indication for lead extraction and the most frequent reason for TLE [6,7]. Due to advances in extraction techniques and tools, TLE has undergone an evolution from a high-risk procedure to a safe and highly successful procedure in many clinical situations [7,8]. Nevertheless, despite the low complication rate, the European Lead Extraction Controlled (ELECTRa) study shows that 38.7% of TLEs were performed under general anaesthesia [9]. The ELECTRa study displayed very low rates of anaesthesia-related complications during TLE, but general anaesthesia is resource intensive and, in general, has higher risk than sedation [9].

General anaesthesia (GA) is defined as the inability for a patient to be awakened, even by painful stimulation, and necessitates intubation. In contrast, patients under deep sedation (DS) will respond purposefully to repeated and painful stimuli, normally without the need for intubation [10]. TLE under conscious sedation (lighter than deep sedation, patients who purposefully respond to verbal or tactile stimulation) alone with diazepam and/or midazolam has been shown to be feasible [11]. DS is common in many other invasive interventions, such as ablation of atrial fibrillation, and has been shown to be safe and feasible [12]. Similar results have been published in CIED implantation procedures, such as ICD and CRT-D implantations [13].

Over the past years, sedation management during cardiac procedures has shifted from anaesthesiologist-directed to cardiologist-directed sedation management. Cardiologist-directed sedation in elective procedures, such as device implantations and catheter ablation, has been reviewed as safe and is recommended during the majority of these procedures [14]. However, this recommendation does not extend to TLE procedures; there are currently no official recommendations for anaesthesia in TLE. The 2018 EHRA expert consensus statement on lead extraction did not give a recommendation due to a paucity of evidence and encouraged further data collection to clarify this aspect [6].

We therefore analysed the safety and feasibility of cardiologist-directed deep sedation during TLE in a single-centre cohort.

## 2. Materials and Methods

### 2.1. Study Population and Data Analysis

Patients undergoing TLE from 2016 to 2019 at the German Heart Centre, Munich were analysed in this trial. Inclusion criteria were indications for TLE, as illustrated in Table 1. Exclusion criteria were age < 18 years, pre-operative cardiogenic shock or intubation/ventilation. During the study period, the standard of care at our institution was DS for TLE. Patient history as well as routine laboratory markers were obtained from every patient. Baseline characteristics are illustrated in Table 1. Sedation protocols from TLE procedures displaying blood pressure, medication administration, sedation time, oxygen saturation, airway management, as well as complications were analysed retrospectively. Sedation-associated complications were defined as transient hypotension (systolic blood pressure < 90 mmHg), persistent hypotension (requiring noradrenaline), bradycardia requiring atropine, non-invasive airway management and unplanned intubation. Impact of DS on patients was analysed based on patient characteristics, TLE indications and periprocedural parameters. The study was approved by the Responsible Ethics Committee (School of Medicine, Technical University of Munich) and conducted according to the principles of the Declaration of Helsinki.

### 2.2. Transvenous Lead Extraction and Sedation Management

We performed all TLEs in an operating room, specialized for CIED procedures. At least one experienced electrophysiologist assisted by an experienced nurse performed the procedure. DS was performed by a specifically trained cardiologist. All cardiologists were trained in advanced cardiac life support and underwent special training in deep sedation for up to 4 weeks by working with a DS-experienced anaesthesiologist or cardiologist. All nurses were trained in basic life support.

We used mepivacaine 1% as local anaesthesia. Fentanyl, midazolam and propofol were used for deep sedation and analgesia. Midazolam and fentanyl were applied as bolus at the beginning of the procedure. Propofol was infused continuously to guarantee sufficient sedation and was adjusted to the depth of sedation. Additional midazolam and fentanyl boluses were applied during the procedure depending on pain and arousal levels. Depth of DS was checked by close monitoring of intraarterial blood pressure, oxygen-saturation, blood gas analyses and by response to voice and tactile stimuli. All patients received two peripheral intravenous catheters (at least 18 G). Catecholamines were administered if persistent hypotension developed. Pacemaker-dependant patients received temporary right ventricular pacing catheters during the procedure.

The active fixation mechanism (if present) was retracted, and simple traction was applied. If simple traction was unsuccessful, a sequential escalation of tools from locking stylets to non-powered and then powered sheaths and snares were used to facilitate the procedure. Continuous on-call support of cardiothoracic surgeons and anaesthesiologists was guaranteed during the whole procedure. If intubation was needed, support of an anaesthesiologist was requested. 

### 2.3. Statistical Analysis

We used IBM SPSS Statistics Version 28 for statistical analysis. Continuous variables were reported as mean ± standard deviation (SD) with 95% confidence interval or as median and inter-quartile range (IQR). Categorical data are presented in absolute numbers and percentage. Comparisons for continuous variables between patient groups were performed by using the Student’s *t*-test in case of normal distribution and the Mann–Whitney U test in abnormally distributed data as well as ANOVA for multiple comparisons. Comparison between categorical data was performed using the Chi squared test. *p* < 0.05 was considered significant.

## 3. Results

### 3.1. Study Population

In total, 341 patients underwent TLE from 2016 to 2019. Of those, eight cases (2.4%) had GA from the beginning of the procedure due to critical conditions such as cardiogenic shock or pre-existing ventilation. Thus, 333 patients (97.7%) underwent TLE with DS. Five patients were excluded due to incomplete data set. Consequently, 328 patients were included in the analysis. Mean age was 65.4 ± 17.1 years, and 70.7% of patients were male. Mean BMI was 27.1 ± 5.2 kg/m^2^. The mean GFR was 68.8 ± 27.0 mL/min. The mean LVEF was 42.6 ± 14.4%. The unplanned presence of an anaesthetist was required in one case (0.3%) in a patient who developed sudden asystole during RV-lead extraction and required cardiopulmonary resuscitation (CPR) as well as emergency intubation.

Indications for TLE were lead dysfunction (45%), lead endocarditis (15%), pocket infection (14%), lead perforation (10%), lead dislocation (7%), vascular complications (5%), patient preference (2%), chronic pain (<1%) and device relocation following radiation (<1%) (Table 1).

### 3.2. Lead Characteristics and TLE Techniques

In total, 562 leads were extracted in this study. Lead extraction was successful in 96.4%. Active fixation pacing leads (48%), passive fixation pacing leads (11%), LV leads (9%), ICD single-coil leads (22%), ICD dual coil leads (6%) as well as S-ICD leads (1%) were included. Dwell time of leads was <1 year in 153 leads (30.1%), 1 to 5 years in 175 leads (34.4%), 5 to 10 years in 141 leads (27.7%), 15–20 years in 24 leads (4.7%) and >20 years in 16 leads (3.1%). Techniques required for TLE: traction and stylet (54.5%), traction and locking stylet (1.76%), unpowered sheaths (10.6%), powered sheaths (32.6%) and snares combined with advanced technique (0.9%).

### 3.3. Medication Dosages during DS

DS was started by a bolus injection of midazolam (mean dose 42.9 ± 26.5 µg/kg bodyweight). Fentanyl (mean dose 0.4 ± 0.6 µg/kg bodyweight) was added in 121 cases (36.9%). Before starting the procedure, local anaesthesia with mepivacaine 1% (mean dose 38.0 ± 19.0 mL) was applied. To maintain DS, a propofol infusion was administered continuously with a flow rate of 3.5 ± 1.2 mg/kg bodyweight/h over an average time of 1.7 ± 0.8 h and was adjusted 2.1 ± 2.1 times during the procedure (Table 1).

**Figure 1:**
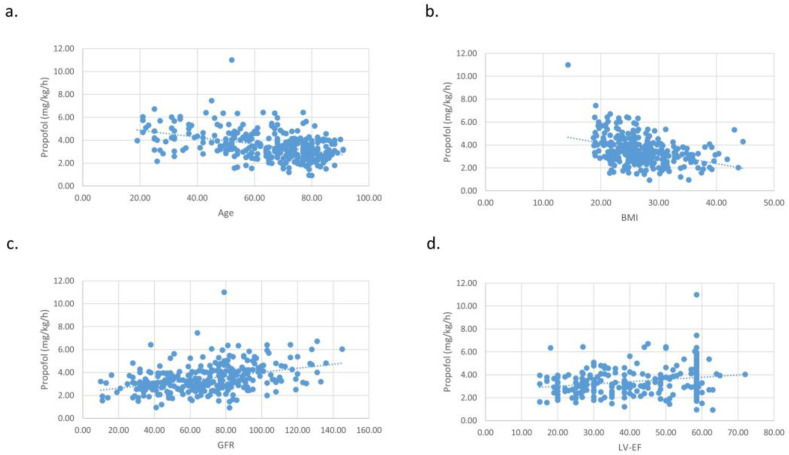
Correlations of the propofol dosage with age (**a**), BMI (**b**), kidney function (GFR) (**c**) and heart function (LV-EF) (**d**). The propofol correlated with heart (LV-EF) and kidney function (GFR) and correlated inversely with age and BMI of the patients.

The propofol and fentanyl dosage correlated with LVEF and GFR and correlated inversely with the age and BMI of the patients. The midazolam dosage also correlated with GFR, but not with LVEF. The midazolam dosage correlated inversely with age and BMI (Table 2, Figure 1).

### 3.4. Deep Sedation-Related Outcomes 

**Figure 2:**
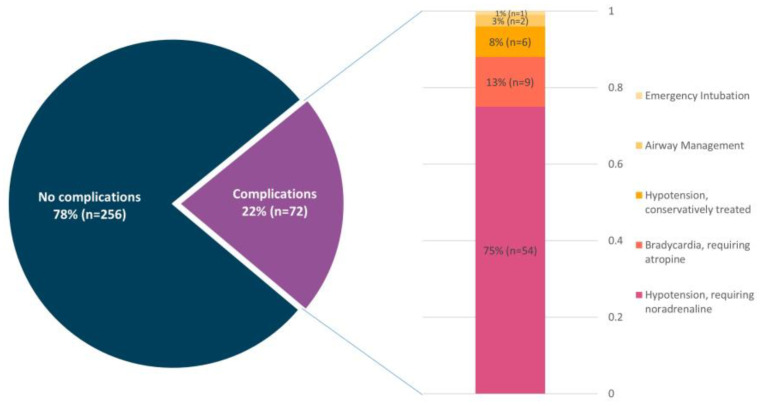
Distribution of sedation-associated complications during TLE. A total of 78% of patients did not suffer any complications. Within complications, the most common complication was hypotension requiring noradrenaline (75%), followed by bradycardia requiring atropine (13%) and conservatively treated hypotension (8%).

Overall, adverse events relating to DS occurred in 22.0% of cases. Adverse events included hypotension requiring noradrenaline administration (16.5%), bradycardia requiring atropine (2.7%), additional non-invasive airway management (0.6%) and unplanned intubation (0.3%). An additional 1.8% of patients developed hypotension managed with fluid administration alone (Figure 2).

The complication rate was above the overall average (22.0%) in patients undergoing TLE due to pocket infection (23.4%, 11/47 patients), lead endocarditis (29.2%, 14/48 patients), lead perforation (36.4%, 12/33 patients) and vascular complication (38.9%, 7/18 patients). Specifically, noradrenaline requirement was significantly higher in patients undergoing TLE due to lead endocarditis (*p* = 0.01) and lead perforation (*p* = 0.05), and it was significantly lower in patients undergoing TLE due to lead failure (*p* = 0.03) (Table 3). Multivariate analysis to identify potential predictors of DS-related complications was undertaken. Lead dwell time (OR 1.13 [1.07–1.20] *p* < 0.01), LV-EF (0.97 [0.94–0.99] *p* < 0.01) and reason for lead extraction (*p* < 0.01) were associated with increased risk of DS-related complications.

We subsequently evaluated the effect of lead dwell time on procedural characteristics and complication rate; there were no significant differences in patients’ baseline characteristics including age, sex, BMI, LV-EF, renal function, or underlying condition leading to CIED implantation (Supplementary Table S1).

An infective indication for device extraction (CIED associated infective endocarditis and generator pocket infections) was more frequent amongst patients with longer lead dwell times; lead dysfunctions and lead perforation were less common indications for extraction in those with longer dwell times. Patients with a lead dwell time > 5 years had a longer procedure (1.6 ± 0.8 h vs. 1.9 ± 0.8 h) and more complications (total complication rate 16.8% vs. 29.4%) driven by bradycardic events. Despite this, there was no significant difference in the dosage of sedation medications or DS-related complications (Supplementary Table S1).

The mean LVEF of patients receiving noradrenaline was lower than those who did not require noradrenaline (38.7 ± 15.0% vs. 43.8 ± 14.1%, *p* = 0.01). Patients with a reduced GFR (61.1 ± 24.5 mL/min vs. 70.9 ± 27.3 mL/min, p=0.01) or an ASA classification > 3 (*p* = 0.01) also had significantly higher complication rates (Table 1).

With regards to the TLE indication, patients undergoing TLE due to lead failure received a higher dose of propofol (3.4 ± 1.2 mg/kg/h vs. 3.7 ± 1.2 mg/kg/h, *p* = 0.02) with a greater number of infusion rate changes (1.9 ± 1.7 vs. 2.4 ± 2.5, *p* = 0.02) compared to remaining patients with sedation-associated complication (Table 4). Patients undergoing TLE due to lead endocarditis had a significantly lower dose of propofol (3.6 ± 1.2 mg/kg/h vs. 3.1 ± 1.3 mg/kg/h, *p* = 0.02) (Table 4). The dose of fentanyl was higher in patients undergoing due to pocket infection (0.3 ± 0.6 µg/kg vs. 0.5 ± 0.8 µg/kg, *p* = 0.02) (Table 4). Patients requiring noradrenaline did not receive higher doses of propofol, fentanyl or midazolam (Table 5).

### 3.5. Procedural TLE Outcomes

In 96.4% of all cases, complete procedural success was achieved. The overall procedural complication rate was 0.9% (n = 3). Two patients developed pericardial tamponade during TLE, and both were treated successfully by pericardiocentesis. The first case was a 75-year-old male who developed pericardial tamponade following removal of the atrial lead. A period of CPR for 30 s was necessary. Intraprocedural pericardiocentesis and noradrenaline were required to stabilise the patient. The whole procedure was performed in DS without requirement to transition to GA.

The second case was a 79-year-old male patient who developed pericardial tamponade after extraction of the RV lead. Treatment was with immediate pericardiocentesis. Again, DS was maintained during the whole procedure.

The third case was a 72-year-old male patient who developed sudden asystole during RV lead extraction. Cardiopulmonary resuscitation (CPR) was commenced, and pericardial tamponade was excluded by echocardiography. Emergency intubation by the anaesthetist, in addition to five minutes of CPR and noradrenaline administration, was required. The case was abandoned. During the removal of the superior vena cava sheath, asystole occurred, and a further 10 mins of CPR was required. Laceration of superior vena cava as well as pulmonary embolism were excluded by means of a CT scan. The retrospective diagnosis was vagally mediated asystole.

## 4. Discussion

Driven by both increasing demand and safety, TLE procedures are being performed much more frequently. Despite the reduced complication rates, the EHRA position paper from 2012 suggests it is advantageous for the patient to be fully anaesthetised in case of possible complications [15]. On the contrary, DS is common in many other electrophysiological interventions such as catheter ablation, pacemaker and ICD implantation [16,17]. GA allows for more physiological control of the patient and facilitates emergent cardiothoracic surgery. However, the need for such surgery is extremely rare and most complications of TLE can be managed without GA, such as the two incidences of tamponade in our cohort. Indications for immediate cardiothoracic surgery include catastrophic cardiac rupture or SVC tears, while other complications (such as pericardial effusions not resolving with pericardiocentesis or proximal vascular damage) usually will allow time for conversion to GA from DS.

The safety of DS for TLE has two dimensions: procedure safety and the safety of the DS itself. We have previously published on the procedure safety of TLE in the modern era and the data in this recent study (0.9% major complication rate) confirm and expand on that safety message [18]. The safety of DS has not been previously evaluated on a large scale.

In our cohort, the rate of any DS-related complication was 22.0%. The majority (83%) of the DS-related complications were hypotension; all of these were successfully and safely managed by the DS directing cardiologist, mostly by conservative management or administering noradrenaline. The definition of hypotension as a DS-related complication should be viewed in the context of the comparable incidence of hypotension requiring inotropic support during GA. Hyman et al. studied DS and GA use in transcatheter aortic valve implantation (TAVI); their analysis demonstrated that the inotropic requirement was more frequent in the GA group compared to the DS group [19]. Thus, the rate of complications in a GA cohort would likely be higher if the same definitions were used to identify complications. Similar results were published in the context of percutaneous mitral valve repair, where the requirement of norepinephrine was significantly higher in GA compared to DS [20].

The requirement for non-invasive airway support (provided by the DS Cardiologist) was 0.6% (n = 2), and only one patient required an anaesthetist to be present for emergency intubation. This highlights that DS TLE procedures rarely require anaesthetic support (in our cohort 0.3%), but it remains essential to have an on-call anaesthetic team in case of rare emergencies. Specifically, patients with longer lead dwell time, depressed LV function and chronic lead complications may require additional caution with DS. This is consistent with previous studies suggesting that the most common adverse events associated with DS were hypotension, bradycardia and hypoxia, all easily manageable by appropriately trained staff [13].

The requirement for inotropic support was not related to the DS medication dose. Inotropic support was required more frequently in patients with an infective indication for extraction. These patients received less propofol, suggesting that it was the underlying pathology that pertained to the risk of hypotension. Patients with local pocket infection did not receive local anaesthesia and had a higher requirement for fentanyl, which may have contributed to a higher rate of hypotension. We observed an association between both depressed LVEF and an infective indication for TLE with the requirement of noradrenaline consistent with previous data [21,22]. These characteristics may be used to identify patients at higher risk. Our findings strongly suggest that those patients with severe cardiac impairment or concurrent septic shock deserve very particular attention while administering DS.

Bode et al. previously evaluated DS in TLE in a study of 220 patients with DS administered by a trained nurse. Compared to Bode et al., patients in our cohort received 76.5% more midazolam, 2.9% more fentanyl and 5.7% less propofol [23]. Patients in our cohort also had a slightly higher requirement for vasopressors compared to Bode et al. (16.5% vs. 11.4%).

In many countries and institutions, GA is a limited resource. This may influence the decision to extract leads for discretionary (non-infective) indications, thus potentially leading to disparity in the level of care delivered. Furthermore, GA is associated with increased requirement for inotropic support compared to DS. This is highly relevant for patients with pre-operative LV impairment, a common finding in patients presenting for lead extraction [19].

On the one hand, concerns have been previously raised about the use of propofol by non-anaesthetists, and this practice is prohibited in many countries [10,24]. On the other hand, non-anaesthetist administration of propofol is already performed frequently in many electrophysiologic and non-electrophysiologic procedures internationally [20,23,25,26,27,28,29,30,31]. Rather than being based on clinical data, this contradictory practice reflects regulatory statements such as the FDA’s: “Propofol should be administered only by persons trained in the administration of general anaesthesia and not involved in the conduct of the surgical/diagnostic procedure”. Our data add to the current cross-speciality evidence suggesting safe use of propofol in this situation.

Importantly, all cardiologists managing DS at our centre undergo dedicated training for a minimum of four weeks. This training facilitates the appropriate knowledge and skillset to perform DS safely. With variation in cardiology training programs between countries, the duration and intensity of this DS management training may need to be more extensive if this approach were to be adopted in other countries. We believe that with specific training, it is reasonable and safe for cardiologists to use propofol in the settling of DS due to the familiarity of managing cardiovascular instability.

Considerations for the use of propofol in short-duration procedures such as cardioversions are different than for longer procedures such as TLE. The pharmacokinetic properties of propofol such as sequestration in adipose tissue become more relevant as procedure length and BMI increase, and this must be taken into consideration for its safe use [32]. The complexity of propofol pharmacokinetics highlights the importance of appropriate training and familiarity with the drug before use in routine practice.

As this was a single-centre retrospective observational study, the patient number was limited. However, our centre is representative of a typical, high-volume extraction centre. Our study did not randomise patients to DS or GA, and thus we were not able to directly compare the two approaches; this could be considered in future studies. Additional patient-focused outcomes such as satisfaction and pain scores would also be of interest in future studies.

## 5. Conclusions

In this study cohort, cardiologist-led DS was shown to be a safe method of sedation during TLE. Complications, predominantly hypotension, were manageable in most cases by a specially trained cardiologist without requiring an anaesthesiologist. We continue to recommend the availability of anaesthetic and surgical assistance in case of severe complications requiring intubation or emergency surgery.

## Figures and Tables

**Table 1:** Comparison of baseline characteristics, TLE indications, procedural data and ASA classification in patients depending on complications.

	Mean ± SD or Count (%)			*p*-Value
	Total	No Complications (n = 256)	Complications (n = 72)	
**Baseline characteristics**				
Age (years)	65.4 ± 17.1	64.7 ± 17.5	68.0 ± 15.6	0.1
Male (n, %)	232 (70.7%)	181 (70.7%)	51 (70.8%)	0.9
BMI (kg/m^2^)	27.1 ± 5.2	27.3 ± 5.3	26.3 ± 4.7	0.2
Heart function (LV-EF in %)	42.6 ± 14.4	43.8 ± 14.1	38.7 ± 15.0	0.01
Kidney function (GFR in ml/min)	68.8 ± 27.0	70.9 ± 27.3	61.1 ± 24.5	0.01
Comorbidities				
Ischemic Cardiomyopathy (n, %)	128	96 (37.5%)	32 (44.4%)	0.3
Dilatative Cardiomyopathy (n, %)	56	41 (16.0%)	15 (20.8%)	0.4
Channelopathy (n, %)	11	9 (3.5%)	2 (2.8%)	0.6
Structural heart disease (n, %)	37	30 (11.7%)	7 (9.7%)	0.8
Congenital heart disease (n, %)	18	16 (6.3%)	2 (2.8%)	0.4
Other heart disease (n, %)	78	64 (25.0%)	14 (19.4%)	0.4
ASA classification				
ASA 1 (n, %)	7	7 (2.7%)	0 (0.0%)	0.2
ASA 2 (n, %)	30	29 (11.3%)	1 (1.4%)	0.01
ASA 3 (n, %)	272	209 (81.6%)	63 (87.5%)	0.3
ASA 4 (n, %)	17	9 (3.5%)	8 (11.1%)	0.01
**TLE indications**				
Pocket infection (n, %)	47	36 (14.1%)	11 (15.3%)	0.8
Lead endocarditis (n, %)	48	34 (13.3%)	14 (19.4%)	0.2
Lead failure (n, %)	148	124 (48.4%)	24 (33.3%)	0.02
Lead dislocation (n, %)	24	20 (7.8%)	4 (5.6%)	0.6
Chronic pain (n, %)	1	1 (0.4%)	0 (0.0%)	0.8
Lead perforation (n, %)	33	21 (8.2%)	12 (16.7%)	0.04
Vascular complication (n, %)	18	11 (4.3%)	7 (9.7%)	0.1
Patients demand (n, %)	8	8 (3.1%)	0 (0.0%)	0.2
System relocation (n, %)	1	1 (0.4%)	0 (0.0%)	0.8
**Procedural data**				
Duration (h)	1.7 ± 0.8	1.6 ± 0.7	2.0 ± 0.8	<0.01
Midazolam (mg/kg)	42.9 ± 26.5	43.2 ± 26.5	42.1 ± 26.5	0.8
Propofol (mg/kg/h)	3.5 ± 1.2	3.6 ± 1.2	3.3 ± 1.1	0.1
Fentanyl (µg/kg)	0.4 ± 0.6	0.3 ± 0.6	0.4 ± 0.8	0.3
Mepivacaine 1% (mL)	38.0 ± 19.0	38.0 ± 18.5	38.1 ± 20.6	0.9
Propofol change rates (n)	2.1 ± 2.1	2.1 ± 2.2	2.4 ± 2.0	0.2

**Table 2:** Correlations of deep sedation medication dosages and age, BMI, heart function (LV-EF) and kidney function (GFR).

	Midazolam (µg/kg)	Fentanyl (µg/kg)	Propofol (mg/kg/h)	Propofol Rate Changes
**Age**				
Pearson’s r	−0.215	−0.236	−0.428	−0.156
*p*-value	<0.001	<0.001	<0.001	0.005
**BMI**				
Pearson’s r	−0.227	−0.108	−0.382	0.079
*p*-value	<0.001	0.059	<0.001	0.173
**Heart function (LV-EF)**				
Pearson’s r	0.078	0.165	0.229	−0.063
*p*-value	0.204	0.007	<0.001	0.300
**Kidney function (GFR)**				
Pearson’s r	0.173	0.188	0.384	0.067
*p*-value	0.003	0.001	<0.001	0.264

Pearson’s r: Pearson’s correlation coefficient.

**Table 3:** Comparison of intraprocedural complications depending on TLE indications.

**Intraprocedural Complication**	**TLE Indication: Lead Endocarditis**		***p*** **-Value**
	**No Lead Endocarditis** **(n = 58)**	**Lead Endocarditis (n = 14)**	
Hypotension, requiring arterenol (n, %)	41 (70.6%)	13 (92.9%)	0.01
Bradycardia requiring atropine (n, %)	8 (13.8%)	1 (0.7%)	0.8
Hypotension (n, %)	6 (10.4%)	0 (0.0%)	0.03
Emergency intubation (n, %)	1 (1.7%)	0 (0.0%)	0.7
Airway management (n, %)	2 (3.4%)	0 (0.0%)	0.6
**Intraprocedural Complication**	**TLE Indication: Lead Perforation**		***p*** **-Value**
	**No Lead Perforation** **(n = 60)**	**Lead Perforation (n = 12)**	
Hypotension requiring arterenol (n, %)	44 (73.3%)	10 (83.3%)	0.05
Bradycardia requiring atropine (n, %)	8 (13.3%)	1 (8.3%)	0.9
Hypotension (n, %)	5 (8.3%)	1 (8.3%)	0.8
Emergency intubation (n, %)	1 (1.7%)	0 (0.0%)	0.7
Airway management (n, %)	2 (3.3%)	0 (0.0%)	0.6
**Intraprocedural Complication**	**TLE Indication: Pocket Infection**		***p*** **-Value**
	**No Pocket Infection** **(n = 61)**	**Pocket Infection (n = 11)**	
Hypotension requiring arterenol(n, %)	45 (73.8%)	9 (81.8%)	0.5
Bradycardia requiring atropine (n, %)	7 (11.5%)	2 (18.1%)	0.5
Hypotension (n, %)	6 (9.8%)	0 (0.0%)	0.6
Emergency intubation (n, %)	1 (1.6%)	0 (0.0%)	0.7
Airway management (n, %)	2 (3.3%)	0 (0.0%)	0.6
**Intraprocedural Complication**	**TLE Indication: Lead Failure**		***p*** **-Value**
	**No Lead Failure** **(n = 48)**	**Lead Failure** **(n = 24)**	
Hypotension requiring arterenol (n, %)	37 (77.1%)	17 (70.8%)	0.03
Bradycardia requiring atropine (n, %)	4 (8.3%)	5 (20.8%)	0.5
Hypotension (n, %)	4 (8.3%)	2 (8.3%)	0.6
Emergency intubation (n, %)	1 (2.1%)	0 (0.0%)	0.4
Airway management (n, %)	2 (4.2%)	0 (0.0%)	0.2
**Intraprocedural Complication**	**TLE Indication: Vascular Complications**		***p*** **-Value**
	**No Vascular Complications** **(n = 65)**	**Vascular Complications** **(n = 7)**	
Hypotension requiring arterenol (n, %)	50 (76.9%)	4 (57.1%)	0.6
Bradycardia requiring atropine (n, %)	9 (13.8%)	0 (0.0%)	0.5
Hypotension (n, %)	4 (6.2%)	2 (28.6%)	0.01
Emergency intubation (n, %)	1 (1.5%)	0 (0.0%)	0.8
Airway management (n, %)	1 (1.5%)	1 (14.3%)	0.01

**Table 4:** Comparison of deep sedation medication regarding TLE indications.

**Medication**	**Total**	**TLE Indication: Lead Endocarditis**		***p*** **-Value**
		**No Lead Endocarditis**	**Lead Endocarditis**	
Midazolam (µg/kg)	42.9 ± 26.5	43.1 ± 25.6	42.0 ± 31.6	0.8
Fentanyl (µg/kg)	0.4 ± 0.6	0.4 ± 0.6	0.2 ± 0.4	0.2
Propofol (mg/kg/h)	3.5 ± 1.2	3.6 ± 1.2	3.1 ± 1.3	0.02
Propofol rate changes (n)	2.1 ± 2.1	2.2 ± 2.2	1.8 ± 1.4	0.2
**Medication**	**Total**	**TLE Indication: Lead Perforation**		***p*** **-Value**
		**No Lead Perforation**	**Lead Perforation**	
Midazolam (µg/kg)	42.9 ± 26.5	42.8 ± 26.9	43.9 ± 22.9	0.8
Fentanyl (µg/kg)	0.4 ± 0.6	0.4 ± 0.6	0.2 ± 0.4	0.3
Propofol (mg/kg/h)	3.5 ± 1.2	3.5 ± 1.2	3.4 ± 1.3	0.6
Propofol rate changes (n)	2.1 ± 2.1	2.2 ± 2.1	1.9 ± 2.1	0.6
**Medication**	**Total**	**TLE Indication: Pocket Infection**		***p*** **-Value**
		**No Pocket Infection**	**Pocket Infection**	
Midazolam (µg/kg)	42.9 ± 26.5	43.2 ± 27.1	41.4 ± 21.9	0.7
Fentanyl (µg/kg)	0.4 ± 0.6	0.3 ± 0.6	0.5 ± 0.8	0.02
Propofol (mg/kg/h)	3.5 ± 1.2	3.5 ± 1.2	3.3 ± 1.1	0.2
Propofol rate changes (n)	2.1 ± 2.1	2.2 ± 2.2	1.8 ± 1.5	0.3
**Medication**	**Total**	**TLE Indication: Lead Failure**		***p*** **-Value**
		**No Lead Failure**	**Lead Failure**	
Midazolam (µg/kg)	42.9 ± 26.5	40.6 ± 24.2	45.6 ± 28.8	0.1
Fentanyl (µg/kg)	0.4 ± 0.6	0.3 ± 0.6	0.4 ± 0.7	0.7
Propofol (mg/kg/h)	3.5 ± 1.2	3.4 ± 1.2	3.7 ± 1.2	0.02
Propofol rate changes (n)	2.1 ± 2.1	1.9 ± 1.7	2.4 ± 2.5	0.02
**Medication**	**Total**	**TLE Indication: Vascular Complications**		***p*** **-Value**
		**No Vascular Complications**	**Vascular Complications**	
Midazolam (µg/kg)	42.9 ± 26.5	43.3 ± 26.6	37.2 ± 23.7	0.3
Fentanyl (µg/kg)	0.4 ± 0.6	0.4 ± 0.6	0.4 ± 0.5	0.9
Propofol (mg/kg/h)	3.5 ± 1.2	3.5 ± 1.2	3.6 ± 1.0	0.8
Propofol rate changes (n)	2.1 ± 2.1	2.1 ± 2.1	1.7 ± 2.1	0.4

**Table 5:** Comparison of deep sedation medication depending on hypotension requiring arterenol.

	Patients Requiring Arterenol (n = 54)	Patients without Arterenol Application (n = 274)	*p*-Value
**Midazolam (µg/kg)**	44.3 ± 25.7	42.6 ± 26.7	0.7
**Fentanyl (µg/kg)**	0.4 ± 0.7	0.3 ± 0.6	0.3
**Propofol (mg/kg/h)**	3.4 ± 1.1	3.5 ± 1.2	0.5
**Propofol rate changes (n)**	2.6 ± 2.1	2.0 ± 2.1	0.08

## Data Availability

Data are available on request.

## Supplementary Materials

The following supporting information can be downloaded at: https://www.mdpi.com/article/10.3390/jcm12154900/s1, Table S1: Comparisons for continuous variables between patient groups were performed by using Kruskal-Wallis Test, comparison between categorical data was performed using the Chi squared test. *p* < 0.05 was considered significant.
