# Supplementary material for: Cardiologist-Directed Sedation Management in Patients Undergoing Transvenous Lead Extraction: A Single-Centre Retrospective Analysis
Source: J Clin Med. 2023 Jul 26;12(15):4900. doi: 10.3390/jcm12154900 (PMC10420171; doi:10.3390/jcm12154900)
Supplement: Supplementary file 1 [file jcm-12-04900-s001.zip › jcm-2432021-supplementary.pdf]

Supplementary Table S1. Comparisons for continuous variables between patient groups were performed by using Kruskal-Wallis Test, comparison between categorical data was performed using the Chi squared test. $p < 0.05$ was considered significant.

	**Group I**	**Group II**	**Group III**	**p-value**
**With lead dwell time**	**<1 year**	**1-5 years**	**>5 years**	
**Number of patients, n**	101	101	126	
Baseline characteristics				
Age (years)	65.2±18.0	65.7±15.8	65.3±1.6	1.0
Male (n, %)	64 (63%)	75 (74%)	93 (73%)	0.2
BMI (kg/m²)	27.0±5.5	27.4±5.3	27.0±5.0	0.8
Heart function (LV-EF %)	43.0±14.5	41.9±14.0	43.0±14.9	1.0
GFR (ml/min)	72.1± 26.7	69.8±25.1	65.2±28.5	0.2
Comorbidities, n (%):				0.6
Ischemic Cardiomyopathy	34 (33.7%)	44 (43.6%)	50 (39.7%)	
Dilatative Cardiomyopathy	20 (19.8%)	17 (16.8%)	19 (15.1%)	
Channelopathy	2 (2.0%)	2 (2.0%)	7 (5.6%)	
Structural heart disease	11 (10.9%)	13 (12.9%)	13 (10.3%)	
Congenital heart disease	4 (4.0%)	5 (5.0%)	9 (7.1%)	
Other heart disease	30 (29.7%)	20 (19.8%)	28 (22.2%)	
TLE indications, n (%)				
Pocket infection	7 (6.9%)	10 (9.9%)	30 (23.8%)	<0.1
Lead endocarditis	5 (5.0%)	19 (18.8%)	24 (19.1%)	<0.01
Lead failure	35 (34.7%)	57 (56.4%)	56 (44.4%)	<0.01
Lead dislocation	19 (18.8%)	2 (2.0%)	3 (2.4%)	<0.0
Chronic pain	0 (0%)	1 (1.0%)	0 (0%)	0.32
Lead perforation	28 (27.2%)	2 (2.0%)	3 (2.4%)	<0.01
Vascular complication	5 (5.0%)	5 (5.0%)	8 (6.3%)	0.86
Patients demand	2 (2.0%)	4 (4.0%)	2 (1.6%)	0.48
System relocation	0 (0%)	1 (1%)	0 (0%)	0.32
Procedural data				
Duration (h)	1.6±0.7	1.6±0,8	1.9±0.8	<.0.01
Midazolam (mg/kg)	42.4±26.4	41.6±17.8	44.5±31.9	0.6
Propofol (mg/kg/h)	3.4±1.1	3.5±1.1	3.6±1.3	0.7
Fentanyl (µg/kg)	0.3±0.6	0.3±0.5	0.5±0.7	0,2
Mepivacaine 1% (ml)	37.1±17.3	40.1±16.6	37.2±21.9	0.2
Propofol change rates (n)	2.1±2.7	2.2±1.7	2.1±1.9	0.3
Complications, n (%)				
Atropine Therapy	0 (0%)	0 (0%)	9 (7.1%)	<0.01
Noradrenaline Therapy	14 (13.9%)	16 (15.8%)	24 (19.0%)	0.57
Hypotension	3 (3.0%)	1 (1.0%)	2 (1.6%)	0.56
Emergency intubation	0 (0%)	0 (0%)	1 (0.8%)	0.45
Airway Management	1 (1.0%)	0 (0%)	1 (0.8%)	0.63

Total	18 (17.8%)	17 (16.8%)	37 (29.4%)	0.04
